# Hotspots and Cutting-Edge Visual Analysis of Digital Museum in China Using Data Mining Technology

**DOI:** 10.1155/2022/7702098

**Published:** 2022-05-25

**Authors:** Yajing Hou, Lijun Xu, Lu Chen

**Affiliations:** Institute of Art and Design, Nanjing Institute of Technology, Nanjing, China

## Abstract

During the last several years, the building and development of digital museums has grown in importance as a study issue of increasing importance. On the other hand, systematic and extensive literature study on digital museums is rare in the academic community throughout the world. This paper employs data mining technology to conduct a comprehensive analysis of the total amount of academic literature, research hotspots, frontiers, and trends in the field of digital museums in China since the beginning of the twenty-first century, including both historical and contemporary data. In this research, the CNIK database and the CiteSpace program are utilized. The findings revealed that the quantity of published literature expanded significantly between 2000 and 2021, with some variations along the way, but that the general growth rate remained consistent. Colleges and universities are the driving force behind academic research in the field of digital museums; research institutes and big museums play a key part in the academic research that is being conducted by digital museums. Cooperation between research institutes, on the other hand, is severely lacking. Furthermore, the advancement of digital technology is an unavoidable byproduct of the efforts to transform the digital museum into a smart museum, as previously said. When it comes to digital museum development in the postepidemic period, the optimization and updating of a user-centered information service platform is the most important step toward long-term success. In order to maintain the richness of Chinese traditional culture while also meeting the expanding cultural requirements of the general public, China's digital museum research has as its ultimate objective the construction of sustainable digital museums that are appropriate for the country's national conditions. The findings also demonstrate that the construction of a Chinese Digital Museum is a study issue with distinct Chinese features that has the potential to contribute to the preservation of Chinese cultural heritage, both tangible and intangible. This research gives insights into the following aspects: researchers and practitioners from across the world will work together to promote a better knowledge of the building and growth of the digital museum in China, among other things.

## 1. Introduction

Since its inception in the 1990s, the digital museum has amassed more than two decades of experience. A global perspective reveals that foreign development is generally quick and comprehensive and that there are several eye-catching building projects that should be studied and promoted throughout the world. Despite the fact that China got off to a late start, the country has achieved many incredible things.

The Library of Congress sponsored the “American Memory” initiative in 1990, which served as the official launchpad for the “Digital Museum” movement. It was in 1995 that the Museum Internet System was officially established in the United States, which integrated many collection information data into the network system, allowing the information of museum collections to transcend the limitations of time and space, and thus signaling the maturation of the digital construction of American museums. Since 2010, several museums have begun to seek the refined services of the target audience, subdivide the target population on the museum website, and adapt popular science information for them in order to continually extend their impact and enhance the attention of users. Since 2011, with the help of powerful digital technology and Internet technology, Google has collaborated with museums around the world to launch the Google art project, which uses Google Street View technology to shoot the real scene inside the museum and equipment with an accuracy of up to 7 billion pixels to shoot famous historical paintings in the museum for global users to enjoy [1]. Google art project uses Google Street View technology to shoot the real scene inside the museum and equipment with an accuracy of up to 7 billion pixels to shoot famous historical paintings in the museum for global users to enjoy. Visitors may choose from more than 18 languages, including English, French, and Japanese, as part of the program. The Canadian Heritage Information Network, or CHIN, has collaborated with major institutions to create the Virtual Museum of Canada, which is now open to the public (VMC). Beyond virtual exhibitions, the website also includes special columns such as interesting games, a teacher center, community memory activities, and online shopping to help bring the digital museum even closer to people's lives. This position in social and cultural service, integrating education and entertainment, has been described as follows: social and cultural service position integrating education and entertainment [2].

The European virtual museum was formally opened to the public in 2008, marking the first anniversary of the museum's creation. It is based on a real museum and incorporates digital assets from the digital library, museum, and archives into one cohesive whole. More than 2000 years of human history have been preserved in digital archives in Europe [3], making them the world's largest repository of historical information. In terms of digital construction, the Louvre Museum in France has broken through the static Internet page and is reshaping the museum website through the use of virtual panoramic technology. Visitors to the website may engage in “virtual tourism” and visit various locations around the museum by using the mouse to move up, down, left, and right through the many rooms [4]. In 2013, the British Museum utilized the crowdsourcing platform to digitize its collections, which was a first for the institution. It completed the tasks that previously required a large number of professionals' long-term efforts and even commercial solutions and it established a new way of working in the field of culture and the Museum [5]. This was accomplished through the joint efforts and cooperation of offline and online, professionals, and volunteers.

Despite the fact that Asian nations implemented museum digitization later than western countries, they were fast to identify a route for developing the digitalization of local museums in accordance with their own growth and national requirements. During 2006, Japan established the “Digital Museum Research Association,” which investigated a mode of collaboration between the government and large corporations that combined the scientific and technological R&D capabilities of large corporations with the cultural relics resource capabilities of museums [6]. As a result of its “borderless” Digital Art Museum, which completely relies on modern digital technology to show art works, it has wowed visitors from all over the world in 2018, providing ample evidence of the success of its digital development strategy.

Taiwan has a head start on the Chinese mainland when it comes to the digital creation of museums. Taiwan's “scientific committee” started the “Digital Museum” initiative in 1998, with the goal of integrating museum materials and creating a museum with local features in order to broaden the meaning of online education [7] in the country. The Natural Science Museum of Taiwan, in collaboration with Jinan University of Taiwan, is putting the idea into action. The “Taiwan collection digitization project” was established in 2001 with the goal of digitizing Taiwan's collections, creating a database, and making it available to the public over the Internet [7]. As a result, the well-known “Imperial Palace digital museum” project in Taipei has been progressing at a quick pace as well. The accomplishments in the areas of collecting, application, and instruction offered useful experience for the Chinese mainland in the area of museum digitization.

The project of “cultural relics survey and database management system” was begun by the China National Cultural Relics Bureau in September 2001, marking the first time that the Chinese mainland has carried out the National Museum digitization effort [8]. The computer network information center of the Chinese Academy of Sciences has built a virtual museum and a China popular scientific Expo based on the database information resources of the Chinese Academy of Sciences, using web technology and multimedia technology. Founded in 1998, it is the first virtual museum group in China, with six display spaces spanning the domains of natural science and social science, respectively. Its digital building knowledge and experience has been widely shared and studied by others. Following that, the Beijing Palace Museum, the Nanjing Museum, and the Dunhuang research center all began working on the digital creation of their own museums. A number of them, such as the Palace Museum and the Dunhuang research institute, have chosen to collaborate with internationally renowned cultural and museum organizations in order to develop, apply, and show Chinese cultural heritage to a wider audience throughout the world. Furthermore, the digital development of Chinese museums does not overlook the importance of education as a basis. To encourage the development of digital resources in colleges and universities, the “action plan for the revitalization of education in the twenty-first century” in China launched the project “construction of online public resources of Modern Distance Education - Construction of University Digital Museum” at the end of 2001 [9]. It was in 2012 that Baidu Encyclopedia collaborated with eight well-known museums in China to launch the Baidu Encyclopedia digital museum, which introduced cultural relics through text and image as well as interpretation audio, animation, and virtual reality, among other methods, greatly improving the user experience and allowing the cultural relics of the museum to come into the public's vision in a more personable, easy to understand, and convenient manner [6, 7].

Through the use of algorithms, data mining is the process of extracting hidden and market-value information from massive amounts of data. The field of data mining technologies is typically associated with computer science and technological fields. Many other technologies, including as statistics, online analysis and processing, information retrieval, machine learning, and so on, can be used to fulfill the aforementioned aims. In data depth mining, the most commonly used approaches are clustering, classification and prediction, and association and deviation detection [10], among others. Generally speaking, the functions performed by data mining technologies are primarily divided into two categories: (1) characterizing specific objects and (2) making appropriate projections [11].

CiteSpace software is utilized for bibliometric analysis in this study to mine the significant documents of digital museums released in China from the beginning of the twenty-first century (from 2000 to 2021). The following issues are addressed in this study, which is conducted in the setting of China: there will be a statistical analysis on the number of academic achievements representing China's digital museum; (2) distribution and cooperation of document quantity analysis and research institutions in different periods; (3) exploration of the evolution of the digital museum from 2001 to 2021, as well as identification of research hotspots; and (4) identification of research frontiers and trends.

## 2. Materials and Methods

The topics covered in this part are primarily four: (1) research tool selection; (2) research data collection; (3) parameter configuration and analysis of the CiteSpace; and (4) an explanation of the primary reference indexes (see Section 1).

### 2.1. Research Tool Selection

Chen Chaomei, professor of computer and information technology at Drexel University, developed the CiteSpace program in 2004 [12]. CiteSpace is citation visual analysis software that allows users to visually analyze citations. With visual approaches, it is capable of objectively processing a huge quantity of scientific literature data and presenting the knowledge structure, law, and distribution included in text materials, resulting in the creation of a “scientific knowledge map” [13]. A wide range of academic study subjects, including library, information, and archives management, management science and engineering, education, sociology, and other fields [14], have been more interested with and applied to the software. The primary reason for using this tool in this study is that it can create a knowledge map of a specific field through visualization functions such as collaboration between authors and institutions, keyword co-occurrence and clustering, emergence, and so on, allowing researchers to explore the development of a subject knowledge field and its research hotspots in greater detail.

### 2.2. Research Data Collection

The CNKI database (China's National Knowledge Infrastructure) was chosen for this study since it is one of the most important and reputable Chinese academic databases in the country. The information utilized in this study was acquired from the CNKI database on February 29, 2022, and it included all papers pertaining to the digital museum from January 2000 to December 2021, which was the time period under consideration. CNKI's database contains the first academic publication on digital museums published in 2000, and this is the year in which the data for this study were originally compiled.

Using the term “Digital Museum” as the subject, this study searched the relevant literature in the CNKI database, yielding a total of 1763 publications. Furthermore, we carefully removed the possibility of contamination from seven unrelated literature and only included peer-reviewed scholarly publications on digital museums in our analysis. Finally, 1756 publications were chosen as the subject of the investigation. During February 2022, CiteSpace, V. 5.8. R1 (https://Citespace.podia.com/download, accessed on 29 February 2022) was used to process all of the selected articles related to the digital museum.

### 2.3. Parameter Setting and Analysis

Before you begin processing the data, you must first configure the settings in CiteSpace. (1) The node type was chosen in accordance with the appropriate analysis; (2) the time slice was set to 2000–2021; (3) the length of each time slice was “2”; (4) the selection criteria were set for the top *N* = 50; and (5) pruning was set as the pathfinder in the selection criteria. The default values have been assigned to the remaining parameters.

Second, CiteSpace is used to evaluate the data from three primary routes once the parameters have been specified in order to address the research difficulties. For the first path, we looked at the quantity of papers published during this time period (as supplied by the CNKI study) and the cooperation network of research institutions, in order to gain a more comprehensive view of the whole research environment. The second approach was to do keyword-based co-occurrence analysis of the data. CiteSpace's co-occurrence analysis generates a map of keyword co-occurrences as well as a map of time-zone co-occurrences. It is possible to obtain the research hotspots of China Digital Museum at different times by doing a thorough study of these two maps. This is the third method, which is known as noun-term burst analysis, and it may be used to demonstrate the quick change of keywords in a short period of time while emphasizing the abrupt shift of keywords [15]. It might also represent the current state of research in this topic, as well as new trends.

### 2.4. Main Metrics Analysis in CiteSpace

Following the completion of CiteSpace according to the specified specifications, three metrics (betweenness centrality, silhouette, and modularity) are primarily utilized to assess the logic of the map that has been produced. When it comes to structural indexes, betweenness centrality is an essential one to consider. It refers to the ratio of the shortest path via a point (connecting two locations in a network) to the total number of shortest path lines between the two points [14]. Nodes having a mediation centrality greater than 0.1 are referred to as key nodes. The presence of purple circles on the map implies that the betweenness centrality is more than 0.1 [16]. The modularity (*Q*) value and the mean silhouette (*S*) value were the two most important variables to assess for the influence of the clustering map. The modularity index of a network is represented by the term modularity. The greater the value of this parameter, the better the clustering outcome of the network will be. The *Q* value ranges between 0 and 1, and a value greater than 0.3 indicates that the split clustering structure is statistically significant. The closer the value is to one, the greater the effectiveness of the clustering effect. The silhouette (*S*) index is used to assess the homogeneity of a network's structure. The closer it is to 1, the more accurately it reflects the homogeneity of the network. When the value is more than 0.5, it is acceptable to conclude that the clustering result is reasonable. Generally speaking, when the *S* value is 0.7, clustering is both efficient and convincing [12].

## 3. Descriptive Statistical Analysis of the Research Literature in the Field of Digital Museum in China

This section discusses two issues: (1) the analysis of the number of papers published in different periods aims to gain a comprehensive understanding of the dynamic distribution of research in this field over the past 20 years; and (2) the analysis of the number of papers published in different periods aims to gain a comprehensive understanding of the dynamic distribution of research in this field over the past 20 years. (2) The cooperation study of research institutions seeks to identify prominent academic institutions as well as the nature of the cooperative connection between research institutions, among other things.

### 3.1. Distribution of the Selected Papers in the Field of Digital Museum

**Figure 1:**
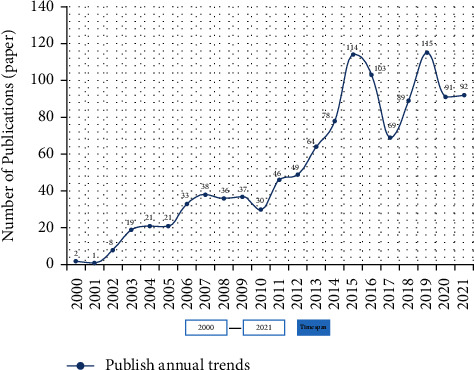
Distribution of selected digital museum papers from 2000 to 2021 in China.

As reported in the yearly analysis report provided in the CNIK database, 1756 papers relating to intangible cultural assets were published between January 2000 and December 2021, representing an average annual publishing rate of 83 publications each year.  Figure 1 depicts the distribution track of the number of selected research articles from 2000 to 2021, as represented by the number of selected research papers. There are three periods that may be identified: the steady development period (2000–2010), the rapid growth period (2010–2015, 2017–2019), and the fluctuation decrease period (2000–2010), (2015–2017).

The number of papers published during the time of stable development, particularly between 2000 and 2002, is extremely modest, accounting for only 0.95 percent of the total number of documents issued during this period. Since 2002, the number of papers published has continuously climbed, with an average of 29 articles being published each year. This demonstrates that relevant researchers have begun to pay attention to the growth of the research field of the digital museum, but that the research field is still in its early stages of development, as demonstrated by the findings of the study. Following that, the number of articles published each year has increased significantly since 2010, reaching its first peak (114) in 2015. This can be attributed to an increase in the number of scholars who have invested in the relevant research field of digital museum, which has received an increasing amount of attention from the academic community in recent years. During the next four years, there was a large fluctuation in the amount of study material published. From 2015 to 2017, the number of papers published declined precipitously, but the number of articles published again increased to reach its second peak (115) in 2019. Each number's trajectory is shown as a horizontal “s” curve, suggesting that there have been considerable changes when compared to preceding periods. From 2015 to 2021, the number of published papers accounted for 58.0 percent of the total, demonstrating that digital museum research is continuously becoming more mature, which may be attributable to the steady establishment of a research system in this subject.

### 3.2. Distribution of Core Institutions in the Field of Digital Museum

**Figure 2:**
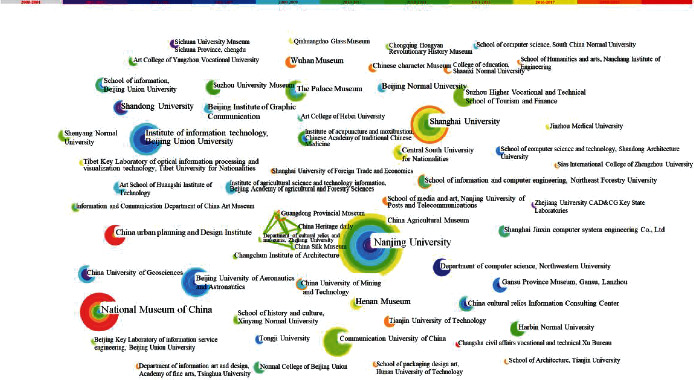
Institution cooperation network map of digital museum research from 2000 to 2021 in China.

The institution network map depicts the spatial distribution of research power in this discipline, and it provides additional information. CiteSpace's cooperative network analysis feature allows researchers in the field of digital museum research to get insight into the network relationships of other institutions in the field. The network link might naturally depict the collaboration between institutions and serve as a point of reference for scientific assessment institutions in the academic domain, according to some researchers.  Figure 2 depicts the distribution network map of digital museum research institutions that was created after the operation was completed. The size of the nodes represents the number of journal papers published by the research institution, and the strength of the connections between nodes represents the intensity of cooperation between different institutions (see text).

In Figure 2, it can be seen that the research sample consists of 310 nodes connected by 39 connections, and the network density is 0.0008, showing that there are many institutions exploring digital museums, but the level of interaction between research institutions is rather low. The fact that the institutions researching digital museums are dispersed and have not created a strong and wide cooperative network link can also be observed.  Figure 2 depicts the formation of an obvious institutional collaboration network, which is comprised of the Guangdong Museum and the Department of cultural relics and museology of Zhejiang University, as seen in the figure. Finally, it is discovered via literature research that the cooperation network has only talked about the issue of “creating a bridge of connection across museum collections” since the 2014 International Museum Day [17], and that it has not engaged in any significant collaboration since then [18]. A collaborative study project involving Zhejiang University, Nanjing University, and four other universities investigated the digital conservation of sports cultural assets.

A further data mining operation is carried out on Figure 2 in order to thoroughly study the accomplishments and collaborative relationships of research institutions, and the top ten research institutions in terms of the number of documents produced are identified, as shown in Table 1. On the list of starting texts, the top three institutions are Nanjing University (number 22), the China National Museum (number 13), and Shanghai University (13). According to Figure 1, the cooperation network association between the top three universities with research results is not statistically significant, demonstrating that even the institutions with high research results do not necessarily have close collaboration with other research institutions. When looking at research units from the perspective of cooperation degree, the cooperation degree of main institutions is low, indicating that, at present, the majority of domestic scholars' research on digital museums is carried out by independent institutions, and there is still a large space for cooperation among institutions, which requires the establishment of a more in-depth cooperation relationship between research institutions from interdisciplinary and cross-regional perspectives.

Table 1 shows the top ten most prolific institutions in the field of China's digital museum, while Table 1 contains the top ten most productive institutions overall. Six universities, two scientific research institutes, and two big museums are among the institutions on the list. This demonstrates that universities are the driving force for scientific investigation in the field of digital museums. Institutions such as research institutions and huge museums play a key role in academic research, which is being driven by digital museums. To provide an example, the State Vital Laboratory of new computer software technology at Nanjing University is tasked with the critical responsibility of conducting research into key technologies for the development of China's digital museum. This is a significant undertaking. The research on digital humanistic storage [19] is being carried out in parallel by Nanjing University and the Tibet Institute for Nationalities, with the goal of laying the theoretical groundwork for the “National Digital Humanistic Infrastructure Construction Project,” which will be launched in the near future. The National Museum of China, as the official representative of China's collection, research, exhibition, and interpretation, is able to fully reflect the country's excellent traditional culture, revolutionary culture, and advanced representative representative material of socialism, among other things. In addition, the national major research and development plan “research and demonstration of key technologies of smart Museum” [20] is being carried out.

## 4. Main Research Hotspots in the Field of Digital Museum in China

Scholars can discover the research hotspot and the evolution of the frontier in this research topic by doing a keyword analysis [12]. In CiteSpace, a keyword analysis is performed in order to create two mappings: co-occurrence knowledge mapping and time-zone view.

### 4.1. Main Research Interests in the Field of Digital Museum: Keywords Co-Occurrence Analysis

**Figure 3:**
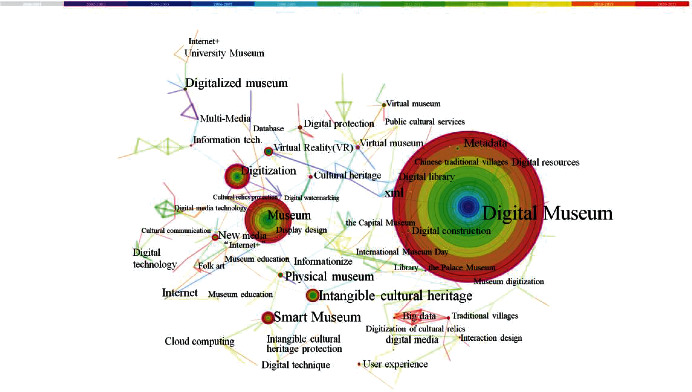
Co-occurrence knowledge map of keywords of the digital museum.

When using CiteSpace, it is possible to do a co-occurrence analysis of terms that appear often in a field to identify important research hotspots [13]. As a result of this procedure, each term may be identified as a potential research hotspot for the digital museum.  Figure 3 depicts the co-occurrence knowledge map of digital museum research terms from 2000 to 2021, as depicted in the previous figure.

We integrated overlapping keywords, such as “digital,” “Museum,” and “Digital Museum,” and removed the search terms that were duplicated. The relevance of the term in the network is reflected in the value of the centrality index of the network map. It is commonly accepted that the keyword with a centrality greater than 0.1 is the more significant keyword. With Figure 2 in mind, the five most frequently occurring keywords of a high school mental nature are as follows: digital museum (frequency = 764; centrality = 0.4); virtual reality (frequency = 60; centrality = 0.28); new media (frequency = 44; centrality = 0.34); and virtual museum (frequency = 30; centrality = 0.6). Cultural heritage (*F* = 28; *C* = 0.36) is a type of heritage. In order to categorize the aforementioned keywords, we searched relevant literature and combined it with a ranking table of high-frequency keywords to create the following three study categories: concept combing, intangible cultural heritage preservation, and information technology. Following that, we will go through each of these three study fields one by one, in conjunction with publications written by prominent domestic researchers.

#### 4.1.1. The Definition Discrimination about Digital Museum

Among the keywords used in definition discrimination are terms such as digital museum, digital library, virtual library, smart library, and smart museum. These rankings are also regularly seen in a variety of research publications, and they are widely debated among academics.

1. *Digital Museum*. The notion of a digital museum is the one that is utilized the most frequently, relative to other concepts. “Digital museum refers to those multimedia digital information institutions that display and exhibit their own cultural relics and specimen collections, use computer digital technology to process, process and sequence, and provide social audiences with browsing and viewing opportunities on the network,” write Zhang and Ning of the China Agricultural Museum. According to some, the digitization of cultural relics or resources, the networking of information transmission, and the promotion of viewing and browsing are the three fundamental characteristics of digital museums [21]. The process of converting a physical museum into a digital museum is thoroughly described in the definition. While digital museums are still confined to “their own cultural relics and specimen collections,” the advancement of technology has allowed them to expand their scope. Some academics argue that digital museums are typically created by museums themselves. Digital websites that provide services for material and intangible cultural heritage are being improved in order to realize the vision of equitable enjoyment of cultural and Expo resources for the entire society and to meet the maximum demand of individuals for cultural and Expo resources [18]. This is in order to realize the vision of equitable enjoyment of cultural and Expo resources for the entire society and to meet the maximum demand of individuals for cultural and Expo resources. Prof. Zheng thinks that the creation of a digital museum is founded on the implementation of museum-related services. In its digital version, it is an information service system for the collection, protection, administration, usage, and transmission of natural and cultural assets in the digital domain. Its presentation and instruction can be carried out in physical museums or across a network [21], depending on the situation.
2. *Digitalized Museum.* It was pointed out by Liu and Zhu that the term “digital museum” has two meanings: “the first refers to the digitization of a physical museum”; and “the second refers to the emergence of virtual museums as a result of the information age's approach, which is not only the result of the digitization of traditional museums, but also the symbol of the modernization of traditional museums [22].” According to some scholars, the term “Digital Museum” is an acronym for “digital museum” in order to separate it from the term “physical museum” [23]. However, some researchers have expressed differing views, claiming that the digital museum is a modern museum following information and digital transformation, which is not the same as a digital museum that exists simply on the Internet [24].
3. *Virtual Museum.* According to Li et al., the virtual museum refers to the re-establishment of the conventional museum through the application of digital technology. There are several components to this digital endeavor. Among the major components of the virtual museum are the fundamental operating platform, the browsing platform, and the administration platform [25]. According to Lv et al., the virtual museum makes use of virtual reality technology to generate simulation models or scenes, which are utilized to convey the overall image of the museum, or situations that were actual in history but have since vanished or are in risk of disappearing completely. Its most distinguishing aspect is its high level of involvement. Visitors can attain the goal of autonomous inquiry learning in the roaming system by engaging with the models and buttons depicted in the figure [26].
4. *Wisdom Museum.* In the same way that the notion of “smart earth” developed, the concept of “smart museum” did as well. It was initially proposed in 2012, and it has since gained popularity. Earlier this year, IBM announced a partnership with the Louvre in Paris, France to optimize the service, operation, and management of the museum by digital technology methods, therefore designating it as the world's first smart Museum. A major project by the State Administration of Cultural Relics of China, titled “feasibility research on the construction of China wisdom Museum” [19], was also launched in the same year. The concept of smart museum building was first proposed by the Chinese government in 2013. A widely accepted definition of smart museum currently exists as follows: “by fully utilizing new generation information technologies, such as the Internet of things, cloud computing, big data, and artificial intelligence in the operation of the museum, we are able to perceive, calculate, and analyze information pertaining to people, things, and activities associated with the museum's operation, realize the intellectualization of museum collection and ensure its protection, display, and dissemination, and conduct research and management of a smart museum.” A comparison was made between the smart museum and conventional museum as well as digital museum, and the “ecological chain under the smart Museum mode” was proposed, which is based on “people,” “things,” and “Museum” [27]. In a systematic manner, the concept of smart Museum was introduced, the similarities and differences between smart Museum and digital museum were discussed, and it was concluded that smart Museum has realized two-way information exchange and effective management of collaborative relationship among “things, numbers, and people” of Digital Museum [28].

#### 4.1.2. Research on Digital Museum in Digital Protection of Intangible Cultural Heritage

Cultural heritage, cultural heritage digitization, cultural relics protection, intangible cultural heritage (protection), traditional villages, and other terms are included in this field.

Human civilization and the crystallization of our predecessors' knowledge are both documented in our cultural legacy, which serves as an essential testimony to the progress of human civilization. Mankind has a responsibility to safeguard and pass it on to future generations. The application of digital technology in the sphere of cultural heritage, according to Song, has the potential to effectively tackle the problems of correct storage, development, and exhibition at this level. To be more specific, the digital filing of three-dimensional modeling and other technologies can exhibit a large number of cultural relics photographs in 360 degrees, allowing the cultural relics to be kept in a more rigorous environment, reducing man-made harm, and extending their service life [29]. It was proposed by Ding and Guo [30] that digital technology represented by augmented reality plays an increasingly important role in cultural heritage protection, and a detailed analysis was conducted of the application of domestic digital technology in cultural heritage protection. Zhou and Yang stepped away from the perspective of technical research and proposed an alternative definition of cultural heritage digitization, which refers to the process of creating relationships through digitization, exploring the core value of cultural heritage, connecting it closely to real life, and assuming today's social and cultural roles [31].

In the United Nations Convention on Cultural Heritage, nonmaterial heritage, culturally significant forms of expression and expressions of noncultural arts and crafts, and the instruments of their protection, are defined as “cultural heritage, nonmaterial objects, performances, and their related groups,” according to the United Nations Educational, Scientific, and Cultural Organization [18, 32]. In 2005, the general office of the State Council issued opinions on strengthening the protection of China's intangible cultural heritage, which stated unequivocally that “we should make true, systematic, and comprehensive records of intangible cultural heritage, in addition to establishing archives and databases.” It was the following year that the “China Intangible Cultural Heritage Network China Intangible Cultural Heritage digital museum” (http://www.ihchina. CN), the world's first worldwide gateway website dedicated to the conservation of China's intangible cultural heritage, was launched.

The specific application of digital technology in the protection, development, and utilization of intangible cultural heritage and its cultural and economic value was developed by Run from the perspectives of museum information technology's investigation and research on intangible cultural heritage, data sorting, data protection, and display [33]. Accordingly, researchers at Tsinghua University, such as Ma et al., investigated the reasons why fundamental digital technologies such as text, picture, audio, and video are still the most commonly used technical applications in intangible cultural heritage digitization, and concentrated on the research and practical results of 3D scanning and reconstruction, virtual reality, augmented reality, motion capture, and other comprehensive technical applications. Although the application of emerging technologies in intangible cultural heritage digitization is still at an exploratory and embryonic stage, it is expected to have a significant impact on future digital protection, particularly in the areas of exhibition and communication [34], as previously stated. Zhou and Yang analyzed and studied the concept and characteristics of intangible cultural heritage digital museum and concluded that the intangible cultural heritage database, the intangible cultural heritage virtual exhibition hall, and the protection and education window are three important components of the intangible cultural heritage Digital Museum [31]. Yan et al. analyzed and studied the concept and characteristics of intangible cultural heritage digital museum, and concluded that the intangible cultural heritage database [35–37]. A large number of other scholars have conducted case studies and strategic analyses on the digital protection of intangible cultural heritage from the perspectives of local characteristic culture, dialect, arts, sports, and so on, thereby broadening the research latitude available in this area.

#### 4.1.3. Research on Information Technology of Digital Museum

Among the terms used to describe the field of information technology are digital technology, virtual reality (VR), augmented reality (AR), new media, and other terms. The development of a digital museum is inextricably linked to the assistance provided by information technology.

According to a survey of the literature, virtual reality technology and augmented reality technology are now two of the most active research areas in the field of digital museum exhibition technology. A high-tech innovation that has emerged in recent years is virtual reality (VR), often known as “spiritual environment technology.” [38] It has the ability to present the audience with an immersive experience through digital reality and virtual digital picture, and it has a unique immersion experience and interactivity. AR technology is a development of virtual reality technology. In this way, the audience can see the virtual model object in the foreground of the real environment, visualize and visualize the abstract learning content, improve the audience's sense of existence as well as their intuition and concentration, and have a more immersive exhibition viewing experience [39, 40]. These technologies are intended to improve the interaction between museum visitors and the museum itself, as well as the overall visitor experience.

The “virtual palace museum” and the “digital Dunhuang” in China are the forerunners in the application of this technology in the field of museum research, providing a cutting-edge learning experience for other museums [40]. For theoretical study purposes, Wang and a large number of other researchers have compiled a list of the relevant technologies of the digital museum. The most significant of them are 3D technology, 360 panorama technology, augmented reality technology, virtual reality technology, and so on. As well as technical assistance for the creation of Digital Museum [41], the development of hardware or technology such as high-definition printers and 3D printing also contributes to its advancement. In addition to the aforementioned technical characteristics, Wu summarized the current web page form of digital museum into four categories: plane web page form, panoramic web page form, 3D cultural relics web page form, and full 3D interactive [42]. This was based on his research into the information technology of digital museum based on the Web.

New media is a type of media that makes use of digital technology, network technology, and mobile communication technology to connect computers, mobile phones, digital television, and other terminals through network channels such as the Internet and mobile communication networks in order to facilitate the exchange and dissemination of user information [43]. A large part of the domestic study on digital museum new media communication is devoted to the investigation of Internet communication routes and challenges. They used unique museum new media communication means such as social media (Wechat and Microblog), webcast, mobile intelligent terminal navigation application, and others to conduct their research [44]. They also compared the communication benefits of the various methods they used. As an example, Hu and Liu developed a development strategy for promoting the Museum of Traditional Chinese Medicine at Beijing University of Traditional Chinese Medicine in order to better meet the development requirements of the new media era. They did this by analyzing and identifying the Museum of Traditional Chinese Medicine's main new media communication channels, as well as identifying the practical problems that existed in the Museum of Traditional Chinese Medicine. The scope of digital museum research has been expanded as a result of this study.

### 4.2. The Evolution of the Research Hotspots in the Field of Digital Museum: Keywords Time-Zone Map Analysis

**Figure 4:**
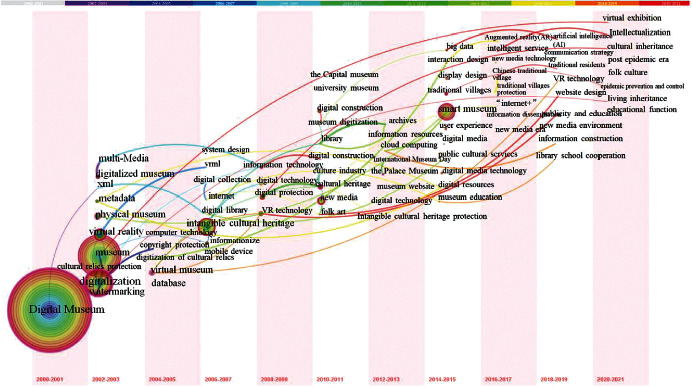
The time-zone view of digital museum from 2000 to 2021 in China.

The development process and Theme Evolution of research subjects in various time periods may be examined by observing the change in keyword clustering time series [13]. This figure (Figure 4) depicts the dynamic process of subject evolution and the layout features of keywords in the field of digital museum, as depicted by the CiteSpace keywords time-zone map. As a result of the increasing number of documents being published in this field year after year (see Figure 1), this study divides the research in the field of digital museum from 2000 to 2021 into four stages and thoroughly discusses the representative articles and significant events that occurred during the contract period.

#### 4.2.1. Stage 1 (2000–2007): Technical Research on the Early Stage of Digital Museum Construction

In the first stage, metadata, XML, VRML, digital watermarking, and cultural relic digitization are the most often used terms, as seen in Figure 4. This demonstrates that relevant technological research in the course of the building of a digital museum was a prominent aspect of the time period in question. It is not difficult to determine the reasons for this by looking back at the development process of China's digital museum from the beginning. As China's first Internet website, the Henan Museum was launched in August 1998, marking the beginning of the country's digitalization of its collections and institutions. The State Administration of Cultural Relics of China launched the project of “cultural relics investigation and database management system construction” in September 2001, marking the first time that a central administrative department has taken on the task of digitizing the National Museum's holdings. The construction and development of the project are inextricably linked to the availability of excellent technical assistance. The development of a digital museum, according to Luo early study, should comprise infrastructure network construction, digitalization of existing items, formation of a database and website, data storage and security, application training for personnel, and other activities [45]. The literature indicates that during this period, Nanjing University, China University of Geosciences, and Zhejiang University's State Key Laboratory of CAD & CG conducted a large number of technical research on XML technology [46], web-based system design [42], and digital watermarking technology [47], and achieved remarkable results, laying the groundwork for the further development of China's digital museum [46].

Figure 4 illustrates that the term “intangible cultural heritage” appears often over this time period. Most importantly, since the general office of the State Council published its recommendations in 2005 on how to strengthen the protection of intangible cultural heritage in China [32], the work of establishing archives and data bases for intangible cultural heritage has been carried out in a comprehensive manner. However, at present moment, research on the digitalization of intangible cultural assets is only in its early stages, with a limited quantity of documents and research tools available. Gradually increasing the depth and range of study in this topic has been done from the year 2010.

#### 4.2.2. Stage 2 (2008–2013): Digital Technology Promotes the Construction of Digital Museum and the Research of Digital Museum in Colleges and Universities

As seen in Figure 4, in the second stage, keywords such as digital technology, information technology, virtual reality technology, and University Museum begin to appear. The following are the primary factors considered in the investigation of its causes. First and foremost, as digital museum technology continues to grow and mature, huge museums are striving to be the first to incorporate digital technology into the digital building of museums, in which virtual reality technology plays an important part. In [38], Yang examined the major technologies that are involved in the application of virtual reality technology to digital museums. Using six virtual reality technologies in digital museums, Zhang et al. conducted an analysis of the technical routes of their application and proposed that, on the basis of not affecting the needs of virtual exhibition and access in digital museums, image-based panoramic virtual reality technology is more suitable for the digital construction of general local museums due to its short development cycle and low development cost [48]. University museums have become high-frequency keywords in part because the State Administration of cultural relics and the Ministry of Education issued a joint notice on strengthening the construction and development of university museums in May 2011, with the goal of strengthening the construction and development of university museums as well as fully developing their role in rejuvenating the country through education and science, learning society, and the consulship system. In the same year, Zhejiang University officially opened the IPv6 site of the China University Digital Museum, which is now available to the public. The digital museum, which is based on the collections of 21 colleges, contains hundreds of thousands of digital items [49] and is constantly expanding. Within a short period of time, university museum research has emerged as a topic of interest in the fields of culture and museum, education, and society.

While these key words were being used, the key words for museums such as the Palace Museum and the Capital Museum were also being used. This was primarily due to the fact that the Capital Museum hosted a Beijing Digital Library seminar in 2013 with the theme of “integration, innovation, and development: the Digital Library in the Constructing of a Cultural Power” [50]. A total of 68 researchers from large museums, universities, and many well-known enterprises from both home and abroad participated in the seminar. They demonstrated through presentations and exhibit the new direction, new achievements, and fresh experience in the field of digital museum construction that have emerged in the last two years, as well as jointly discussed museum construction and provided new ideas and method for building a digital museum. The Beijing Association of Science and Technology, the Beijing Municipal Bureau of Cultural Relics, and the Beijing Municipal Commission of Economy and Information Technology came together in 2005 to form the Symposium on Science and Technology. It is held every two years and has played a significant role in the establishment of China's digital museum, which is now in its third year.

#### 4.2.3. Stage 3 (2014–2019): Research on Digital Museum in New Media Environment

It was during this time period that smart museums, smart services, new media, digital media, and high-frequency phrases and keywords relating to “design” appeared on the Internet. As can be observed, the wisdom museum has emerged as a new research center throughout this time period. The primary reason for this is that, after more than 10 years of building and expansion, the China Digital Museum has amassed an extensive collection of successful examples and research findings. The State Administration for Cultural Relics of China organized the important research topic “feasibility study on the building of China smart Museum” [19] in 2012, and the concept of smart museum construction was first proposed in 2013. As part of its national wisdom Museum pilot program, the State Administration of cultural treasures designated seven museums as the initial pilot units in 2014. Since then, an increasing number of academics have made investments in the study of this topic. Since 2018, smart museum research has emerged as a prominent topic in the museum sector, with the number of papers published in 2019 more than double the number of articles released the previous year. For the most part, the study is concerned with the concept analysis of smart museums [19, 26, 27], associated technologies [51, 52], case studies at home and abroad [53, 54], and the development trend [55, 56], among other things.

It was in 2015 that Chinese Premier Keqiang Li introduced the China Internet Plus Action Plan [57], which was first included in the government work report. On the one hand, the Chinese government premier introduced the notion of “Internet plus” for the first time in his two-year report, which was released in December. New media, as defined by Liu and others, is a new media form that is produced by the mix of conventional media with developing technologies based on the Internet, i.e., “traditional media Plus Internet.” In Digital Museums, new media technologies such as big data and cloud computing, mobile Internet technology and social network technology, flexible display technology and wearable devices, and holographic technology are prominent examples of new media technologies. According to some, new media technology is not just a method of communication, but also a mode of experiencing the world around us. It increases the number of visitors to the digital museum while also providing a higher-quality user experience. It is not difficult to conclude from a survey of the literature that academics' focus has steadily changed from the early stages of museum web design and development [58] to the investigation of interactive design based on new technologies. Specifically, the interactive design of virtual reality technology [59, 60] and mobile client [61] is the focus of the current study.

#### 4.2.4. Stage 4 (2020-2021): Research on Digital Museum in the Postepidemic Era

Postepidemic era, educational purpose, folk culture, and living inheritance are some of the keywords that appear often throughout this time period (Figure 4). In this age, a new COVID-19 “hot postepidemic era” was forming, mostly as a result of the advent of the new crown pneumonia outbreak in late 2019, which was the cause of the new epidemic. At several points during the epidemic's emergency prevention and control in the first half of 2020, public cultural service locations were temporarily shuttered, and offline cultural experiences were put on hold. Several local public cultural institutions have actively changed their service methods and implemented the “cloud” mode in order to meet the spiritual and cultural needs of the public, which includes the ability to seek knowledge and pleasure online without having to leave the comfort of their own homes [62]. The availability of Internet information services is particularly significant in this situation. Construction of online service platforms is moving at a faster pace, which is the general tendency. Because of this, experts have turned their attention to the study of the existing state of digital services provided by cultural and museum institutions, as well as the debate of the specific path for service upgrading, in the aftermath of the pandemic [63].

## 5. Research Frontiers and Trends in the Field of Digital Museum in China

Table 2 displays the phrases that have been used most frequently in digital museum research in China over the last 20 years, with a total of 53 keywords. By analyzing the data, it has been discovered that the five keywords of user experience (strength = 5.29), traditional village (strength = 4.98), smart museum (strength = 4.92), big data (strength = 3.59), and digital protection (strength = 3.28) represent the research frontiers of digital museum research in China in the recent years. The following will be contrasted and discussed in conjunction with a sample body of the literature with a high citation rate.

### 5.1. Research on User Experience Design of Digital Museum

In recent years, as digital technology has progressed and matured, the research direction of the digital museum has gradually shifted away from simple technical research and toward research on user experience. This shift is intended to provide visitors with a more humanized human-computer interaction while also improving the overall experience of visiting and learning. The use of design methodologies to create the display, web page, and mobile client of a digital museum has been a research hotspot in this topic in recent years, particularly from the standpoint of user experience. Interface design [64], exhibition design [61], interaction design [61], and experience design [38] are some of the design research approaches that have been developed. Immersive interface design, experience design, and user experience assessment are just a few of the topics that have emerged as the current study trends.

In his paper, Wang argues that the notions of user experience and user journey are two fundamental concepts in the concept of experience design. The notion of experience design may be used to guide the building of a digital museum, which can assist the museum in better understanding the needs of users and reducing the psychological gap between the museum and the audience to the greatest extent feasible, says the museum. On the one hand, using the network benefits of a digital museum, a virtuous cycle of “sharing receiving resharing” of knowledge may be generated among visitors, which can be used to pique the interest of future visitors in browsing by stimulating their browsing excitement. To some extent, digital museums may be considered to be cooperative endeavors involving the merging of technology with art, and the digital collection of information and culture can be considered to be a significant benchmark for the advancement and optimization of the museum sector in general [65]. Noteworthy is the suggestion made by Fan [66] that the future development of digital museums should not only be user-centered but should also take into account the efficacy of cultural information distribution. Consequently, he employs the coding and decoding theory of information transmission, along with the user interview technique, to develop the user experience evaluation index and user experience assessment model for the digital museum. By doing this study, we are able to close a research gap in the assessment process for user experience in the digital museum.

### 5.2. Research on the Protection of Traditional Villages by Digital Museum

Earlier this year, China's Ministry of Housing and Urban-Rural Development officially began construction on the Digital Museum of Chinese Traditional Villages, with the goal of displaying excellent Chinese traditional villages on a digital platform, thus serving as both a “stage for publicizing Chinese traditional villages to the world” and a “window for the world to understand Chinese agricultural civilization.” The CPC Central Committee and the State Council issued the Strategic Plan for Rural Revitalization (2018–2022) in 2018, which defined traditional villages as “characteristic protected villages” and proposed to effectively protect the material and intangible heritage of traditional villages [67]. The plan was approved by the CPC Central Committee and the State Council in 2018. Throughout the years, study into the digital preservation of traditional villages has steadily gained in importance, and it continues to be a hot topic in the field of digital museum research.

The digital preservation of traditional villages can be accomplished in two ways: first, by archiving, displaying, and publicizing villages in the form of a digital museum; second, by assisting traditional villages in fine management, planning prediction, protection, and development status monitoring through digital means. Also being investigated is the unique application of digital technology in traditional village protection, which is now under development. Recent technologies include man-machine tilt photography, lidar scanning, offline sensors, large data platforms, 3D printing, 3D synthesis technology platforms, artificial intelligence, and others. The following are some examples: practice has shown that Guangzhou University has investigated the combination of “three-dimensional modeling + oblique photography + traditional villages” and has successfully realized the digital presentation and publicity of traditional villages through the new digital process of three-dimensional modeling, online platform construction, cultural and creative industry, and new media promotion. A platform for architectural heritage monitoring developed by Beijing Architecture University has the capability of not only realizing traditional architectural monitoring, but also carrying out restoration prejudgment and simulated restoration [68]. For example, when it comes to the construction of a traditional village digital museum, Song and Zhao proposed that the next task should be to solve the efficiency of spatial data retrieval and the ability of real-time analysis of spatial data so that users can have a more process user experience [69]. Cao and Wu redesigned the information architecture, information presentation, and information dissemination in the digitization of cultural heritage at the Traditional Village Museum, in order to address the problem of information understanding in the digitization of traditional villages [70]. They did so in accordance with the principles of information and interaction design, which they developed.

### 5.3. Development Trend of Future Digital Museum: Smart Museum

However, despite the fact that the concept of a smart museum was first proposed in 2014, the actual building and development of a smart museum in China is still in its early stages. Current intelligent museum architecture depends heavily on a number of developing technologies, including big data, cloud computing, and artificial intelligence [19], in order to actualize the intelligent working mode with the Internet serving as the medium. Since then, the research viewpoint offered by Smart Museums has expanded significantly. In particular, the research from the perspective of public service, as well as the research on the top-level design method of smart museums and the construction of modern smart museum systems, which has emerged as the research frontier in this field, is deserving of special attention and consideration.

In Li's opinion, the smart museum is a new information transmission mechanism developed between people, things, and data that can be used to better understand the world. It also represents a step forward in the preservation of museum collections and public services on all sides of the spectrum. Li conducted a thorough investigation of the process that occurs before, during, and after users browse the smart Museum, and he examined the smart Museum's implementation methods from the standpoint of public service [56]. The operation and maintenance management of the smart museum, according to Zhong and Zhang, is considered a system project. He points out that the smart museum is a subsystem project of the overall system project. He concludes by saying that a research method for top-level design of smart museums was also proposed for the first time by him at the same time. He pointed out that the top-level design of the smart museum is the overall design for the development form and progression path for a digitally enabled museum, which is dependent not only on information technology, but also on a research concept and a research method [19]. In the opinion of Bai S., the wisdom museum may be considered a closed-loop control system that integrates dynamic information collecting, intelligent processing, intelligent control, and analytical feedback. In her remarks, she pointed out that, in order to develop and expand the smart museum system, it is necessary to first establish data specifications and standards, then build the smart service and operating platform of the smart museum, and finally establish standardized operation and maintenance processes [71].

### 5.4. Application of Big Data Technology in Digital Museum

2015 saw the release of the State Council's action plan for promoting the development of big data, which stated that we should encourage the development and application of big data, accelerate the opening and sharing of data, promote resource integration, improve management ability, improve the big data security system, and deepen the security pillar. This demonstrates that the development and deployment of big data technology has advanced to the level of national policy in China [72]. China's Digital Museum has been doing research into big data technologies for some years, with the majority of its efforts concentrating on the preservation of intangible cultural treasures and the development of smart museums.

According to Yang when it comes to intangible cultural heritage protection, we can use big data's unstructured characteristics to develop local intangible cultural heritage management systems, build intangible cultural heritage big data platforms, realize multidimensional communication of intangible cultural heritage, and help the public understand *t*. The omnibus thinking method of big data, in addition, may be utilized to forecast and appraise the communication form and future development trend of intangible cultural assets in a given location [73]. The smart museum, which integrates the Internet of Things, big data, and “cloud computing” technology, can effectively carry out data analysis and processing, allowing the museum's services to be more intelligent and systematic as a result of this combination. At the moment, the use of big data technologies in the management and maintenance of smart museums is being considered. Network information technology and Internet of things technology, as well as real-time monitoring systems and intelligent agent terminal technology [71], are some of the most recent innovations to emerge.

### 5.5. Research on Digital Protection in Digital Museum

When Dr. Zhao of Shandong University wrote his doctoral dissertation “Research on the protection and development of historical and cultural resources under digital survival” in 2014, he went into great detail about how important digital technology is to the protection of China's historical and cultural resources. Dr. Zhao is a professor of history and cultural resources at Shandong University. His research has revealed that, in addition to three-dimensional technology and virtual reality technology, digital image processing technology, multimedia technology, digital content management and publishing technology, three-dimensional scanning technology, and network technology are all essential for the protection of cultural resources [74]. Following a review of the literature, it is shown that the preservation of intangible heritage, cultural heritage, and traditional villages, all of which have been described above, have been the study frontiers of digital protection in the last three years.

The author, after examining the current state of China's intangible cultural heritage digital protection, proposed that in order to ensure the long-term development of China's intangible cultural heritage digital protection, it is also necessary to improve database construction, enrich the communication mode of digital protection, and most importantly to strengthen the intellectual property protection of intangible cultural heritage digitization [36]. Sun et al. [75–77] put forward new perspectives, proposed the application of relevant theories of public management to countermeasures to solve the problem of digitization of cultural heritage, and constructed a New Trinity protection and inheritance model of “government, society, and market” of cultural heritage, which has implications for sustainable development in the context of digital cultural heritage protection. A fresh research viewpoint for the digital safeguarding of cultural assets is also provided by this point of view.

## 6. Discussion

Following the results of the CNKI's literature review, it can be determined that the number of papers dealing with the issue of digital museum rose fast between 2000 and 2021. Despite the fact that the number of papers fell for a little period of time, it quickly grew again. This demonstrates that academics have maintained a significant interest in this topic over the years. Researchers are mostly found at academic institutions, research institutes, and museums, among other places. Universities are the driving force behind academic research in the field of digital museums; research institutes and big museums play a key role in academic research that is facilitated by digital museums. Unfortunately, there is little collaboration across research institutions, and just a few colleges have engaged in collaborative research efforts to far. Intangible cultural heritage, including cultural heritage and traditional villages, is a hot topic in digital museum research, and digital protection of intangible cultural assets, including cultural heritage and traditional villages, is a research frontier. The debate of local situations, in particular, has long been the primary focus of scholars' deliberations. It can be shown that the digital protection of intangible cultural assets is the top priority of the literary research conducted by China's digital museum and that this is supported by the findings of the research.

According to the findings of this study, the emergence of research hotspots in digital museums is primarily influenced by national policies, digital technology, and the requirements of the public. Chinese society and culture are characterized by a top-down social and cultural framework, and government policy supervision and support are believed to be the most important factors in promoting its development. There is no question that information technology has aided the advancement of Chinese digital museum research, and the debate over the practical use of important technologies has emerged as a popular issue among academics. In addition, the advancement of digital technology is an unavoidable consequence of the efforts made to advance the growth of the digital museum into a smart museum. It is worth emphasizing that the postepidemic age has catapulted the field of digital museum research to a new level of sophistication. It is both a difficulty and an opportunity for the development of China's digital museum that the virus has had such a devastating impact. During this time period, the investigation of the unique path of service platform development and service upgrading has been a popular topic of discussion.

It is fascinating, in terms of the research frontier and trend, to investigate the digital museum from the viewpoint of the user experience and to employ the design technique in order to better understand the digital museum, which has drawn the attention of a significant number of scholars. Thus, research on Digital Museum in China has evolved from purely technical to people-oriented and service experience focused research, in line with the overall trend of international research on Digital Museum. It also demonstrates that the perspective and methods of research in this field in China are constantly evolving. Moreover, the researchers who conducted the study discovered that the digital preservation of traditional villages has been gaining more and more attention in recent years, which is sufficient evidence that the Chinese government has elevated Rural Revitalization to a major strategic position. It may be predicted that the research fervor in this sector will continue for a lengthy period of time in the foreseeable future. Furthermore, big data has emerged as a new research frontier for the digital museum, demonstrating that big data technology is a significant driving force for the development of the digital museum, particularly in the digital protection of intangible cultural heritage and the construction of smart museums. Finally, in accordance with the findings of the study hotspots, smart museum is an unavoidable trend in the development of the digital museum in the future.

## 7. Conclusions

The purpose of this study is to give scholars in the area of Chinese digital museum with a quantitative examination of the literature in this subject, in order to get a more in-depth understanding of the growth and evolution of the field of Chinese digital museum during the past 20 years (2000–2021). The findings of the study are mostly congruent with those of other researchers working in the same field of study. The exception is that this study argues that the establishment of a Chinese Digital Museum comprises a research issue with Chinese characteristics for the conservation of Chinese cultural heritage and intangible cultural heritage, and that this is a research topic with Chinese characteristics. In addition, new media technology is a critical component in the construction of a digital museum's infrastructure. The digital museum benefits from not just the utilization of this significant modern technological tool, but also from the high-quality user experience that it gives. It is particularly important in this postepidemic period to optimize and upgrade user-centered information service platforms since this is the only way to ensure the long-term viability of the digital museum. Finally, this study concludes that the ultimate goal of the research on China's digital museum is to find a way to ensure the long-term development of digital museums that is appropriate for China's national conditions, in order to preserve the diversity of Chinese traditional culture while also meeting the growing cultural needs of the general public.

## Figures and Tables

**Table 1:** The ten most productive research institutions in digital museum in China.

Rank	Institution	Year began	Number of papers	Cooperation degree
1	Nanjing University	2002	22	3
2	National Museum of China	2006	13	0
3	Shanghai University	2012	13	1
4	Institute of Information Technology, Beijing Union University	2004	11	0
5	Beijing University of Aeronautics and Astronautics	2003	10	0
6	Communication University of China	2012	10	1
7	Suzhou Higher Vocational and Technical School of Tourism and Finance	2014	7	0
8	China Urban Planning and Design Institute	2019	7	0
9	The Palace Museum	2003	7	0
10	Department of Computer Science, Northwestern University	2004	6	0

**Table 2:** The burst terms in digital museum research in China.
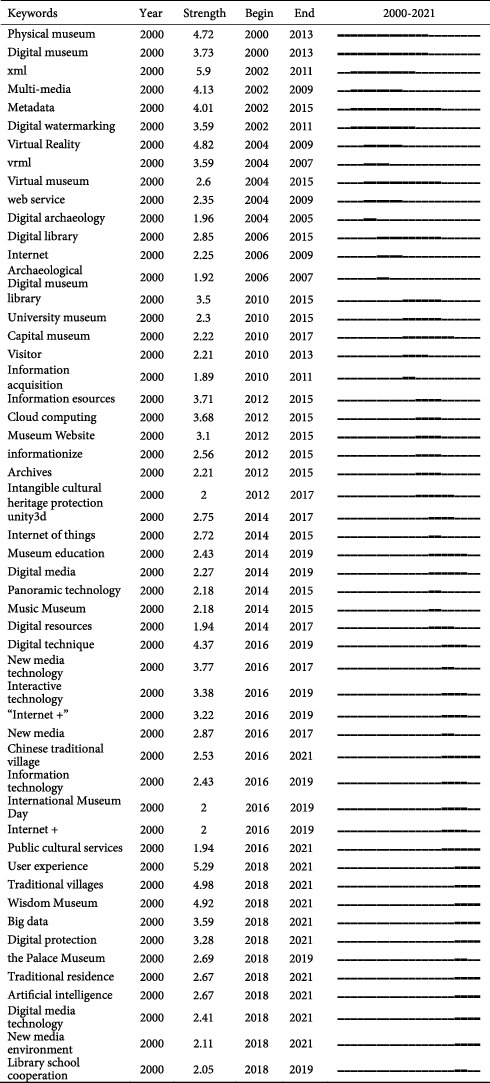

## Data Availability

The datasets used to support the findings of this study are available from the corresponding author upon request.
